# Democratic classroom management and school satisfaction: the serial mediating roles of trust in teacher and school engagement

**DOI:** 10.3389/fpsyg.2026.1862954

**Published:** 2026-07-03

**Authors:** Metin Kırbaç, Onur Balı, Meltem Yurtçu, Ramazan Özkul

**Affiliations:** 1 Faculty of Education, Inonu Universitesi, Malatya, Türkiye; 2 Faculty of Education, Agri Ibrahim Cecen Universitesi, Ağrı, Türkiye; 3 Faculty of Education, Harran University, Şanlıurfa, Türkiye

**Keywords:** democratic classroom management, school engagement, school satisfaction, serial mediation, trust in teacher

## Abstract

**Introduction:**

The present study explores the direct and indirect relationships between democratic classroom management and school satisfaction among students, with trust in teacher and school engagement as serial mediators.

**Methods:**

A correlational research design was employed and data were collected from a sample of 800 secondary school students enrolled in grades 5 to 8 in Türkiye.

**Results:**

Mediation analysis was conducted, and the results indicated that democratic classroom management was a significant predictor of trust in teacher and school engagement but not of school satisfaction. Trust in teacher was a significant predictor of school engagement and school satisfaction, and school engagement was the strongest direct predictor of school satisfaction. Most importantly, democratic classroom management had a significant indirect effect on school satisfaction through both trust in teacher and school engagement, which supported the serial mediation model. The results show that democratic classroom practices positively affect students’ trust in teachers, and this, in turn, leads to higher school engagement and finally to increased school satisfaction.

**Discussion:**

The current study contributes to the existing literature by examining both interpersonal and contextual variables simultaneously in one serial mediation model and by shedding light on the underlying psychological processes through which democratic classroom environments affect students’ satisfaction with school.

## Introduction

In the past decades educational research has undergone a radical change, changing its focus from the negative aspects of education to positive aspects and concepts (Zullig et al., 2011). This shift has brought positive psychology concepts – such as well-being, engagement, resilience, and life and school satisfaction – more into educational research. School satisfaction reflects students’ overall evaluations of experiences regarding their school life. Specifically, early work on school satisfaction mostly focused on indicators of school quality such as school size, teacher-student ratio, leadership style, or school cleanliness (Baker and Maupin, 2009). Apart from school-based indicators, individual factors of students such as gender, race, grades, test scores, or psychological factors were also the focus of researchers (Huebner et al., 2000; Huebner and Alderman, 1993; Okun et al., 1990). However, students’ satisfaction with school also depends on their interactions within the school and on contextual factors. Contextual factors such as school climate, physical and social environment of school, and teacher-student relationship affect the students’ interactions and perceptions within the school, thereby shaping their satisfaction (Coelho and Dell’Aglio, 2019; Suldo and Huebner, 2006; Telef et al., 2015; Zullig et al., 2011).

The importance of school satisfaction derives from its associations with both academic and psychosocial outcomes. Students with higher levels of school satisfaction tend to have higher levels of academic achievement, engagement, and self-esteem, while lower levels of school satisfaction are associated with higher levels of psychological symptoms (Gutiérrez et al., 2017; Huebner and Gilman, 2006). Similarly, students who are satisfied with their school life are less likely to have problematic behaviors (Black et al., 2010). Previous studies have also demonstrated that school satisfaction constitutes a key role for understanding children’s and adolescents’ general life satisfaction and their perceptions of life quality (Huebner et al., 2012). Considering the amount of time that students spent in school, school satisfaction also contributes to their general life satisfaction. From this perspective, it can be highlighted as a factor contributing to both students’ academic functioning and general well-being.

The issue of disengagement and dissatisfaction continues to be a major concern, particularly due to its long-term negative effects on students’ well-being (Dei et al., 1997; Wang et al., 2017). In a yearly report by ACT (Australian Capital Territory), the proportion of students satisfied with their school decreased from 71 to 65% between 2020 and 2024, with the lowest satisfaction observed in Combined schools (54%) (Australian Capital Territory Government, 2021, 2024). Similarly, parental satisfaction declined from 83 to 78% over the same period, again reaching its lowest level in Combined schools (66%) (Australian Capital Territory Government, 2021, 2024). According to a nationwide report of students aged 11–18 in Türkiye, approximately 20% reported experiencing bullying at school and being treated poorly by teachers, while 15% reported that they did not feel safe at school, which in turn undermines students’ engagement and satisfaction (Uyan-Semerci et al., 2024). However, in democratic schools, problems caused by disengagement (e.g., absenteeism, tardiness, and unfinished homework) were found to be relatively low (Pellerin, 2005). Democratic behaviors of teachers strengthen teacher-student interactions, which in turn build trust (Peras and Bezjak, 2025). Also, student-teacher trust supports school engagement and well-being (Diastu et al., 2023). In this context, trust in teacher and school engagement can be theorized as two mediator variables in the relationship of democratic classroom management (DCM) and school satisfaction. Moreover, a comprehensive understanding of school satisfaction and its antecedents is essential to promote academic functioning and positive development of students, and to design effective interventions aimed at supporting students’ success in school and beyond.

**Figure 1:**
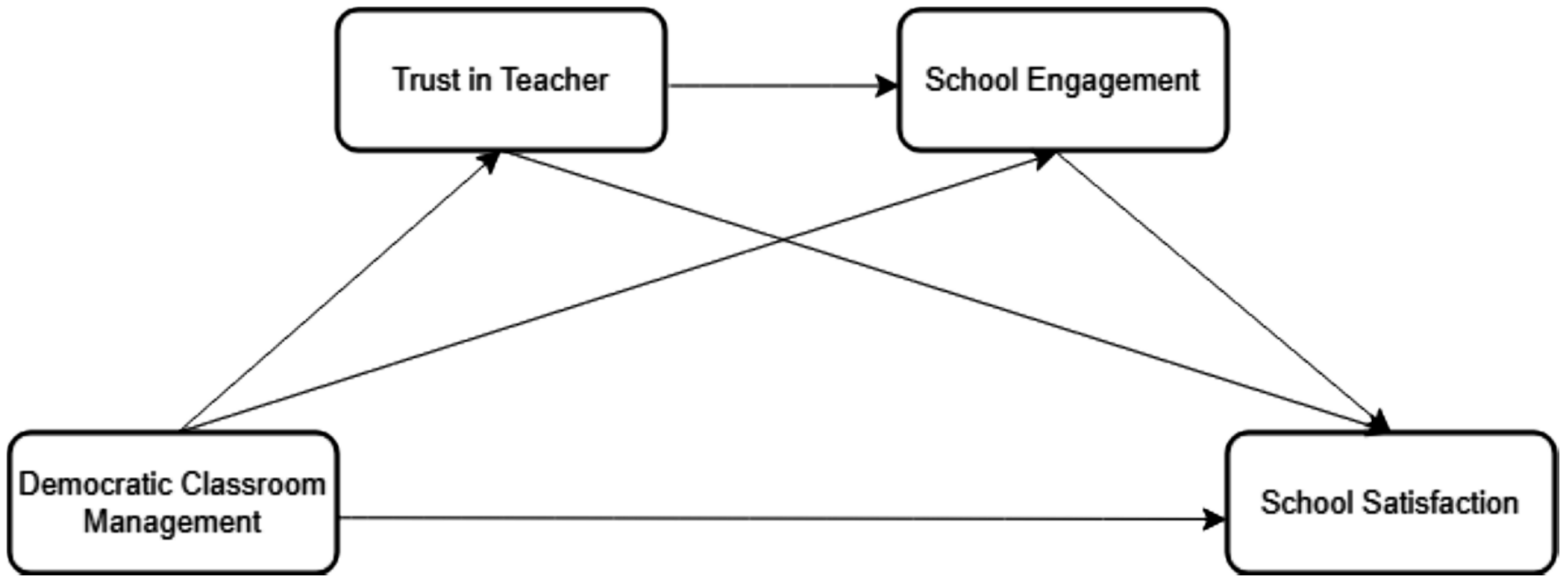
Conceptual model.

The present study employs a serial mediation analysis while investigating the role of trust in teacher and school engagement in the association between DCM and school satisfaction. Investigating students’ school satisfaction can contribute to our understanding of the school life of students. In order to thoroughly understand the school satisfaction in students and provide supportive practices, it is important to consider the underlying mechanism. Despite a growing body of research, relatively less attention has been paid to the determinants that foster students’ school satisfaction (Kim and Kim, 2013). This study adds to existing literature on school satisfaction in two ways. First, it employs a serial mediational approach while analyzing the relationship between DCM and school satisfaction. Second, to our knowledge, this is the first study investigating the effects of both interpersonal (Trust in Teacher) and contextual factors (DCM, School Engagement, and School Satisfaction) together on school satisfaction (see Figure 1).

### Conceptual framework

The present study is built on existing literature on school satisfaction, suggesting that interpersonal and contextual factors in school can promote students’ school satisfaction (Bubic and Goreta, 2015; Lam et al., 2018; Vidic, 2021). Also, Self-Determination Theory (SDT) suggests that contextual resources (DCM) satisfy students’ basic psychological needs and promote respect in the classroom (Ryan and Deci, 2000). Students’ respectful interactions develop trust in teachers, and according to the Social Exchange Theory (SET), trust-based interactions enhance students’ engagement (Bryk and Schneider, 2002; Reeve, 2006). In the context of Türkiye, in which centralized exams occupy a central role, the education system promotes competitiveness among students. Students frequently experience high levels of anxiety and stress due to academic performance expectations in such a competitive culture, which in turn negatively affects their school engagement and school satisfaction (Bali, 2021; Kilit et al., 2020; Vidic, 2021). However, according to existing research, SDT, and SET, democratic classrooms may play a supporting role in fostering trust in teacher, enhancing student engagement, and finally contributing to school satisfaction. In this regard, we proposed a serial mediating model to support the school satisfaction of students. The following sections define the abovementioned concepts and suggest theoretical and empirical bases for the proposed associations in the study.

#### Democratic classroom management

Effective education is more likely to occur in well-managed classrooms (Marzano and Marzano, 2003). Management style of the teacher is a vital element affecting the quality of teaching and learning. Broadly defined, classroom management style refers to the level of teacher participation with students and the type of control strategies implemented by teachers (Burden, 2020). It shapes all kinds of actions of teachers while managing academic, behavioral, social, and emotional aspects of the classroom (Evertson and Weinstein, 2011). Effective classroom management facilitates teaching and learning, and it is associated with better academic, behavioral, and social outcomes among students (Korpershoek et al., 2016). Management style is closely connected with classroom climate, student behavior in the classroom, teacher-student relationships, and student achievement (Kirbac, 2019).

There are several ways to conceptualize classroom management styles. For instance, an established theory that is based on the concept of parenting styles developed by Baumrind (1991) describes four classroom management types: neglectful, indulgent, authoritarian, and authoritative, depending on control and warmth dimensions. Authoritative classroom management is characterized by a combination of high warmth and high control. This method is positively linked with student performance, socioemotional development, and engagement (Kloo et al., 2023; Walker, 2009). Another theory offers four management approaches: autocratic, authoritative, democratic, and laissez-faire, where the democratic classroom management highlights the involvement of students in decision-making processes such as setting class rules and developing self-discipline (Graham, 2018). Among these styles, democratic management relies on students’ engagement in management and requires teachers’ guidance and facilitation of students’ active participation in the classroom (Özkul, 2025). Teachers promote students’ self-discipline and set behavioral standards for them, creating a safe and supportive climate for all students while delegating authority to students (Graham, 2018; Gürbüz et al., 2026; McDonald, 2019). Democratic teachers expect and guide students to take responsibility for their actions, which in turn fosters their self-regulation, self-esteem, and independence. It values and trusts students’ actions while cultivating a classroom climate and a teacher-student relationship (Özkul and Dönmez, 2023; Reeve and Cheon, 2021). Positive relationships between teachers and students help students to form secure and trusting bonds with their teacher (Kremer, 2010). In a research by Djigic and Stojiljkovic (2011), the interactionist approach (sharing the authority of the teacher with students) was found to be associated with better classroom climate and higher school achievement. Moreover, in democratic school settings, problems of absenteeism and tardiness occur less frequently (Pellerin, 2005).

Despite the considerable potential of DCM in enhancing trust in teachers, school engagement, and school satisfaction, to the best of our knowledge, no research has examined the links between these variables within a single model. For instance, Peras and Bezjak (2025) claimed that a democratic classroom culture strengthens student-teacher relationships and builds trust. In a research by Vidic (2021), it was found that a supportive classroom climate increases students’ engagement with school and fosters school satisfaction.

#### Trust in teacher

Trust is commonly defined as one’s willingness to be vulnerable to others based on the belief that the other party is “benevolent, reliable, competent, honest, and open” (Hoy and Tschannen-Moran, 1999). In a similar formulation, Baier (1986) describes trust as reliance on others’ competence, coupled with their willingness to care for rather than harm. As trust supports communicative exchange, greater trust increases individuals’ likelihood of sharing ideas and feelings openly (Tschannen-Moran and Hoy, 2000). In the school context, trust is not only a sign of well-functioning schools but also at the center of the teaching and learning process, given that classroom activities are basically built upon teacher-student interaction (Tschannen-Moran and Hoy, 2000). Therefore, trust constitutes a core element of healthy teacher-student relationships and supportive learning environments, providing the foundation for cooperation and collaboration in classrooms (Platz, 2021; Tschannen-Moran et al., 2006).

It is crucial to emphasize that the context for the formation of trust between students and teachers is heterogeneous. Studies indicate that children from lower socioeconomic status and other marginalized communities may face unique obstacles when establishing trust with their teachers (Lee et al., 2019; Tordön et al., 2021). It is important to understand what impacts the perception of certain classroom activities within marginalized communities.

Schools consist of networks of social exchange among their stakeholders (Bryk and Schneider, 2002). In this environment, daily interactions with respect and fairness cultivate trust between the teacher-student relationship (Tschannen-Moran and Hoy, 2000). Similarly, supportive climates with clear norms, opportunities for participation, and a sense of safety foster trust when teachers are perceived to be predictable and just (Mitchell et al., 2018). Trust in teacher develops when students engage and appreciate their teachers, however it is lost if students are resistant, distracted, or unresponsive against teachers (Sklar and McMahon, 2019). Previous research on trust has shown that higher levels of trust are associated with better academic and social outcomes. Higher levels of trust boost students’ academic optimism and performance (Bevel and Mitchell, 2012; Goddard et al., 2001; Tschannen-Moran, 2004). Moreover it supports collaboration in school, leads to healthier school climates, and strengthen students’ identification with school (Adams and Forsyth, 2007; Mitchell et al., 2018; Tarter and Hoy, 2004). Moreover, according to the Social Exchange Theory, trust-based interactions with teachers in a supportive classroom are expected to enhance students’ engagement (Bryk and Schneider, 2002; Emerson, 1976). Also in a study by Diastu et al. (2023), student-teacher trust was found as a positive predictor of school well-being and engagement.

#### School engagement

School engagement has received growing attention from researchers over the past decades, as it represents a critical factor in students’ academic and psychosocial development. As interest in school engagement has increased, various definitions have been proposed (Fredricks et al., 2004; Jimerson et al., 2003). Engagement is widely defined as students’ involvement, connection, and commitment to academic and social activities in school (Li and Lerner, 2013). It is often conceptualized as a multidimensional construct comprising cognitive, affective, and behavioral components (Fredricks et al., 2004).

Students’ perceptions of school experiences significantly contribute to their active engagement. When teaching and learning practices are considered valuable and meaningful, students are more engaged in school-related activities (Gürbüz et al., 2026). Furthermore, social relationships, particularly those with teachers, peers, and families, serve as major determinants of engagement. In a systematic review by Martins et al. (2022), more than half of the research on school engagement has focused on the role of teachers, classmates, or parents. Perceived teacher support and teacher encouragement enhance students’ engagement in schoolwork, whereas negative interactions and perceptions regarding teachers and teachers’ feedback lead to disengagement (Parsons, 2016).

Engagement is not only important for daily school functioning but also for long-term developmental outcomes. While school engagement studies heavily highlight the importance of academic outcomes, relatively little attention has been directed toward its social, emotional, and psychological benefits (Li, 2011). High levels of school engagement are linked to positive academic achievement, reduced risk of dropout and behavioral problems (Appleton et al., 2008; Kirbac, 2019). On the contrary, low engagement is associated with negative academic and behavioral outcomes, including substance use and involvement in criminal activities (McNeely and Falci, 2004). Baltatescu and Cernea-Radu (2025) found a positive relationship between school engagement and school satisfaction. Moreover, Gutiérrez et al. (2017) found school engagement as one of the positive predictors of school satisfaction.

#### School satisfaction

School satisfaction has increasingly attracted attention of scholars in the field of education in recent years. As students spend a substantial proportion of their time at school, school satisfaction emerges as one of the most salient domains of satisfaction. It reflects students’ evaluations of and their overall satisfaction with their school life and experiences. It is a domain-specific facet of students’ life satisfaction and subjective well-being, which reflects their critical judgments regarding the quality of school life. In the school context, many factors affect students’ satisfaction. Consistent with the Self-Determination Theory, safe, caring, fair, and autonomy-supportive classrooms support students’ satisfaction in school (Reeve and Cheon, 2021; Ryan and Deci, 2000). According to Lodi et al. (2019), quality of school services, relations with classmates, and study habits shape students’ feelings of satisfaction. Furthermore, it is associated with school connectedness, perceived academic support in school, and the school’s physical and social environment (Zullig et al., 2011). It is also related to key aspects of students’ functioning, including their health, happiness, well-being, and behavioral problems (Horanicova et al., 2022; Telef, 2021; Whitley et al., 2012; Yam, 2022).

Students with higher school satisfaction are happier at school and have greater psychological well-being (Yam, 2022). School satisfaction is also a significant predictor of student happiness, with higher school satisfaction associated with greater happiness in students (Telef, 2021). While Pedditzi (2024) mentions that a low level of school satisfaction leads to increased intentions of school dropout in the secondary school context. In a study by Whitley et al. (2012), higher levels of dissatisfaction in school are associated with academic, behavioral, and adaptation problems. Horanicova et al. (2022) found that students who are unsatisfied in school are more likely to skip school, get involved in fights, feel hopeless, and have more health-related complaints. From this perspective, school satisfaction is related to both students’ academic functioning and their everyday life.

### Mediated effects of trust in teacher and school engagement on the relationship between DCM and school satisfaction

Significant progress has been made in this field, both theoretically and empirically, but there are still some gaps. First, although there are many associations among DCM and student outcomes studied so far, the process of how the psychological mechanism through which democratic classroom management contributes to students’ satisfaction via their relationships and motivations has not been tested yet. Second, previous studies have investigated trust in teachers, engagement in schools, and school satisfaction separately, but no studies have considered them all together.

This study was based on Self-Determination and Social Exchange Theory. We conceptualized DCM as a contextual resource that satisfies students’ basic psychological needs and promotes fairness and respect (Reeve, 2006; Ryan and Deci, 2000). In such a classroom context, students develop trust in their teachers (Bryk and Schneider, 2002). From the Social Exchange Theory perspective, trust-based interactions with teachers within a supportive classroom context are expected to enhance students’ engagement (Bryk and Schneider, 2002; Emerson, 1976). As a result of positive classroom and school experiences, students’ overall school satisfaction, a domain-specific cognitive appraisal of school life, is also expected to increase. From this point of view, we model DCM as an antecedent that supports trust in teacher and school engagement, which in turn fosters school satisfaction.

In parallel with this theoretical framework, empirical evidence suggests that DCM, trust in teacher, school engagement, and school satisfaction were associated (Bubic and Goreta, 2015; Lam et al., 2018; Reeve and Jang, 2006; Vidic, 2021). Democratic teachers and classrooms support students’ academic and psychological functioning (Reeve and Jang, 2006). Peras and Bezjak (2025) mention that a democratic school and classroom culture strengthen student-teacher relationships and build trust. Positive relationships with teachers support trust when students engage and appreciate their teachers (Sklar and McMahon, 2019). In a study by Diastu et al. (2023), student-teacher trust was found as a positive predictor of school well-being and engagement. In addition, supportive behaviors of teachers also contribute to the students’ school satisfaction (Bubic and Goreta, 2015; Lam et al., 2018). Ahmad and Said (2014) found that DCM positively predicts students’ engagement, and teachers’ social competence and antisocial behavior as a moderator in this relationship. In a study by Vidic (2021), positive and supportive classroom climate predicts students’ engagement (*β* = 0.50), which in turn enhances school satisfaction. Vidic (2021) found that school engagement has a full mediating role in the relationship between positive classroom environment and school satisfaction.

Although the antecedents of school satisfaction have attracted increasing attention in recent years, there is still much unexplored territory in terms of the role of classroom-level variables in affecting students’ emotional evaluations of their schooling experience. In particular, there is a need for empirical evidence concerning the role that democratic classroom management (DCM) plays in fostering school satisfaction, either directly or indirectly via relational and motivational processes. This study aims to address the following general research question: Through which psychological processes does democratic classroom management contribute to school satisfaction? More specifically, the following research questions were put forward:

*RQ1*. To what extent does DCM predict trust in teacher, school engagement, and school satisfaction among students?

*RQ2*. Does trust in teacher mediate the association between DCM and school engagement?

*RQ3*. Do trust in teacher and school engagement sequentially mediate the relationship between DCM and school satisfaction?

## Method

### Research design

The present study is quantitative in nature and designed using a cross-sectional correlational model. A correlational model is a study conducted to determine the relationships between two or more variables (Fraenkel et al., 2012). In this study, a serial mediational model was conducted with the mediating roles of school engagement and trust in teachers, between DCM and school satisfaction. The conceptual model of the study is presented below in Figure 1.

### Participants

The participants of the study were recruited from secondary schools in the central districts of Malatya, Türkiye. Power analysis was performed with the GPower software to determine the minimum sample size of the study. Data were collected from 825 students using a convenience sampling method. This number was calculated using the GPower analysis, and the collected data exceeded the calculated value. Following the assessment of normality, cases with missing or invalid responses were removed, yielding a final sample of 800 students. The sample of the present study consisted of 800 students in grades 5, 6, 7, and 8 attending secondary schools in the central districts of Malatya province, Türkiye. Among them 386 were female (48.3%) and 414 were male (51.7%). In terms of grade level, 164 (20.5%) of the participants were in the 5th grade, 247 (30.9%) in the 6th grade, 247 (30.9%) in the 7th grade, and 142 (17.8%) in the 8th grade.

### Instruments

#### Classroom management styles scale

Classroom Management Styles Scale is a 17-item scale used to assess classroom management perceptions of students (Kirbac, 2019). It consists of two subscales, namely Democratic Classroom Management Style (DCMS) and Authoritarian Classroom Management Style, and each can be used separately. The 10-item DCMS was used to assess democratic classroom management perceptions of students. Items (e.g., “My teachers establish classroom rules collaboratively with students”) are rated on a five-point Likert scale from 1 (*strongly disagree*) to 5 (*strongly agree*). The total score ranges between 10 to 50 points. The higher the score, the higher the students’ perceptions of democratic classroom management regarding their teacher. The internal consistency coefficient (Cronbach’s Alpha) of the scale was 0.89 (Kirbac, 2019). In the present study, the internal consistency coefficient (Cronbach’s Alpha) was calculated as 0.91. Furthermore, an examination of the CFA (confirmatory factor analysis) results for the scale reveals that *χ*^2^/df = 2.433, RMSEA = 0.04, SRMR = 0.02, CFI = 0.96, TLI = 0.95 and GFI = 0.96.

#### Trust in teacher scale (TTS)

The 12-item TTS (Adams et al., 2009; Turkish version: Özer and Tül, 2014) was used to assess students’ trust in teachers. Items (e.g., “Teachers in our school are honest with students”) are rated on a five-point Likert scale from 1 (*never*) to 5 (*always*). The total score ranges between 12 to 60 points. The higher the score, the higher the students’ trust in teacher. The internal consistency coefficient (Cronbach’s Alpha) of the scale was 0.88 for the secondary school students sample (Özer and Tül, 2014). In the present study, the internal consistency coefficient (Cronbach’s Alpha) was calculated as 0.89. The scale is unidimensional; an examination of the CFA results reveals that *χ*^2^/df = 1.44, RMSEA = 0.06, SRMR = 0.02, CFI = 0.98, TLI = 0.99 and GFI = 0.98.

#### School engagement scale (SES)

The 17-item SES (Kirbac, 2019) was used to assess school engagement. Items (e.g., “I comply with school rules,” “I feel happy while at school”) are rated on a five-point Likert scale from 1 (*strongly disagree*) to 5 (*strongly agree*). The total score ranges between 17 and 85 with higher scores indicating higher levels of school engagement. The internal consistency coefficient (Cronbach’s Alpha) of the scale was 0.88 (Kirbac, 2019). In the present study, the internal consistency coefficient (Cronbach’s Alpha) was calculated as 0.90. The scale consists of three sub-dimensions: cognitive, behavioral and emotional. The results of the CFA show that *χ*^2^/df = 3.74, RMSEA = 0.060, SRMR = 0.024, CFI = 0.93, TLI = 0.96 and GFI = 0.94.

#### School satisfaction scale (SSS)

The 6-item SSS (Randolph et al., 2009; Turkish version: Telef, 2014) was used to assess school satisfaction of students. Items (e.g., “School days are nice,” “Learning makes me happy”) are rated on a five-point Likert scale from 1 (*strongly disagree*) to 5 (*strongly agree*). The total score ranges between 6 to 30 points. The higher the score, the higher the students’ school satisfaction. The internal consistency coefficient (Cronbach’s Alpha) of the scale was 0.89 (Telef, 2014). In the present study, the internal consistency coefficient (Cronbach’s Alpha) was calculated as 0.83. The scale is unidimensional; the CFA results show that *χ*^2^/df = 3.45, RMSEA = 0.06, SRMR = 0.01, CFI = 0.99, TLI = 0.99 and GFI = 0.98.

### Procedure and ethics

Ethical approval for the present study was obtained from İnonu University Social and Human Sciences Ethics Board (Approval Date and No: 06.11.2025-E.667663). After obtaining ethical approval, participants were informed about the study in their schools, where data were collected by the researchers. Informed consent was obtained from all participants in the study. Permission was granted by the parents before the data collection process began. A total of 825 questionnaires were gathered and 25 of them were excluded due to incomplete responses (more than 3 missing items) and straight-lining in all measures. After the exclusions, 800 valid questionnaires were used in the analysis, yielding a response rate of 97%.

### Data analysis (analytical approach)

Four steps have been taken in analyzing data. In the first step, the descriptive statistics (mean, standard deviation, skewness, and kurtosis) of all study constructs were calculated. In the second step, Pearson correlation tests were performed to analyze the bivariate relationships between DCM, trust in teacher, school engagement, and school satisfaction. Convergent and discriminant validities of the study measurement model were evaluated by computing AVE, CR, and the HTMT ratio in the third step. In the fourth step, serial multiple mediation was tested using Structural Equation Modeling with maximum likelihood estimation and using Hayes Model 6 with 5,000 bootstrap samples, and 95% confidence intervals were evaluated (Hayes, 2017). Gender and grade level were taken as control variables. Descriptive statistics and correlations were calculated using SPSS version 26.0, while JAMOVI 2.6.25 and SmartPLS 4.1.1.5 were used for mediation analysis and SEM (structural equation modeling).

### Validity and reliability of instruments

The Average Variance Extracted (AVE) value was examined to calculate convergent validity. AVE values above 0.50 indicate that convergent validity is achieved. Furthermore, to determine whether convergent validity and construct reliability were achieved, the Composite Reliability (CR) value was also calculated, and it was checked whether it was higher than the AVE values and above 0.60 (Fornell and Larcker, 1981; Hair et al., 2021; Yaşlıoğlu, 2017). The results are presented in Table 1.

**Table 1:** Results of convergent validity (AVE, CR).

Variables	AVE	CR
DCM	0.563	0.764
TT	0.467	0.793
SE	0.411	0.873
SS	0.552	0.602

DCM, democratic classroom management; TT, trust in teacher; SE, school engagement; SS, school satisfaction.

Composite reliability (CR) values of all measurement tools are above 0.6, indicating a good level of reliability (Bacon et al., 1995). The Cronbach’s Alpha coefficients calculated to assess the internal consistency of the scales ranged from 0.83 to 0.91. The AVE values calculated for convergent validity are seen to range between 0.41 and 0.56, falling below the recommended value of 0.50 (Fornell and Larcker, 1981; Hair et al., 2021). According to Fornell and Larcker (1981), the extracted average variance may be a more conservative estimate of the validity of the measurement model, and “based solely on CR (composite reliability), the researcher may conclude that the convergent validity of the structure is adequate even if more than 50% of the variance is due to error” (p. 46). Since the composite reliability of the four structures is well above the recommended level and the Cronbach Alpha values are quite good, the internal reliability of the measurement items is acceptable. Furthermore, it is seen that the condition that CR values must be greater than AVE values, another criterion for convergent validity, is also met (Yaşlıoğlu, 2017).

Divergent validity was calculated to determine whether the scale structures were distinct from one another. Divergent validity was measured by examining both whether the square roots of the AVE values were greater than the correlation coefficients between the scales and the Heterotrait–Monotrait correlation (HTMT) values. Correlation and AVE square root values are presented in Table 2, while HTMT values are presented in Table 3.

**Table 2:** Results of divergent validity (AVE square root).

Variables	DCM	TT	SE	SS
DCM	0.750			
TT	0.715	0.683		
SE	0.686	0.650	0.641	
SS	0.554	0.596	0.688	0.742

DCM, democratic classroom management; TT, trust in teacher; SE, school engagement; SS, school satisfaction.

**Table 3:** Descriptives and correlations.

Variables	1	2	3	4	5	6
1. Gender	–					
2. Grade	−0.01	–				
3. DCM	0.04	−0.24**	–			
4. TT	0.04	−0.27**	0.72**	–		
5. SE	0.03	−0.27**	0.69**	0.65**	–	
6. SS	0.08*	−0.23**	0.55**	0.60**	0.69**	–
Skewness	–	–	−0.20	−0.87	−0.74	−0.79
Kurtosis	–	–	−0.92	0.66	0.63	0.77
Mean (SD)	–	–	3.14 (1.01)	3.82 (0.81)	3.69 (0.80)	3.82 (0.98)

DCM, democratic classroom management; TT, trust in teacher; SE, school engagement; SS, school satisfaction. ^*^p < 0.05, ^**^p < 0.01.

Table 2 shows that the AVE square root values range from 0.68 to 0.75, while the correlation values between scales range from 0.55 to 0.71. Since some AVE square root values are lower than the correlation values between scales (Fornell and Larcker, 1981), Heterotrait–Monotrait correlation values were also examined.

As shown in Table 4, all HTMT values are below 0.90, indicating that the measurement model demonstrates discriminant validity (Henseler et al., 2015).

**Table 4:** Results of divergent validity (HTMT ratio).

Variables	DCM	TT	SE	SS
DCM	1			
TT	0.793	1		
SE	0.748	0.715	1	
SS	0.640	0.695	0.786	1

DCM, democratic classroom management; TT, trust in teacher; SE, school engagement; SS, school satisfaction.

### Common method bias

Both statistical and procedural approaches can be used to address common method bias resulting from single-source data collection. One such statistical method is Harman’s single factor method (Harman, 1967). Exploratory factor analysis was performed including all variables within the scope of the research. If a single factor emerges from the factor analysis or if a general factor explains a large portion of the covariance, then common method variance is considered to be present (Podsakoff et al., 2003). As a result of the exploratory factor analysis, 29 factors with eigenvalues greater than 1 emerged. The first factor accounted for 35.82% of the total variance, suggesting that common method bias was unlikely to seriously distort the results (Hui et al., 2025).

## Results

### Descriptive statistics

Descriptive statistics and correlations between democratic classroom management, trust in teacher, school engagement, and school satisfaction are presented in Table 3. The results show significant positive relationships between all variables.

As shown in Table 3, data normality was confirmed prior to analysis, with Kurtosis and Skewness values within the acceptable range of ±1. Furthermore, as all correlations were below 0.80, indicating there was no multicollinearity (Tabachnick and Fidell, 2007).

Students’ perceptions of DCM were found to be significantly and positively correlated with Trust in Teachers (TT) (*r* = 0.72, *p* < 0.01), indicating a strong relationship. Similarly, DCM was significantly and positively associated with School Engagement (SE) (*r* = 0.69, *p* < 0.01) and School Satisfaction (SS) (*r* = 0.55, *p* < 0.01), both reflecting moderate relationships approaching a high level. These findings suggest that as teachers adopt a more democratic approach to classroom management, students’ trust in teachers, their engagement with school, and their overall school satisfaction tend to increase. Furthermore, Trust in Teachers (TT) was found to be significantly and positively correlated with School Engagement (SE) (*r* = 0.65, *p* < 0.01), indicating a moderate-to-high relationship, and with School Satisfaction (SS) (*r* = 0.60, *p* < 0.01), indicating a moderate relationship. This implies that higher levels of trust in teachers are associated with increased student engagement and satisfaction. Finally, a significant and positive relationship was observed between School Engagement (SE) and School Satisfaction (SS) (*r* = 0.69, *p* < 0.01), reflecting a moderate-to-high correlation. Accordingly, as students’ engagement with school increases, their level of school satisfaction also increases.

### Measurement model

Within the scope of the research, multiple alternative models to the existing model were tested. For this purpose, confirmatory factor analysis (CFA) was performed. When examining the goodness-of-fit values of the models, it was observed that the four-factor model performed better than the three-, two-, and single-factor models (*χ*^2^/df = 3.16, *p* < 0.001 RMSEA = 0.05, SRMR = 0.05, CFI = 0.88, TLI = 0.88). In this case, it was concluded that the four-factor model is the most appropriate model for the research questions (see Table 5).

**Table 5:** Alternative test results for the study variables (*n* = 800).

Variables	*χ* ^2^	df	*χ*^2^/df	CFI	TLI	RMSEA	SRMR
Four-factor (original)	2,953	935	3.16	0.882	0.875	0.052	0.046
Three-factor (TT and SE merged)	3,923	939	4.18	0.825	0.816	0.063	0.055
Two-factor (TT, SE and SS merged)	4,368	942	4.63	0.799	0.789	0.067	0.058
One-factor (all constructs merged)	4,990	943	5.29	0.672	0.651	0.083	0.109

TT, trust in teacher; SE, school engagement; SS, school satisfaction.

### Research questions analysis

**Figure 2:**
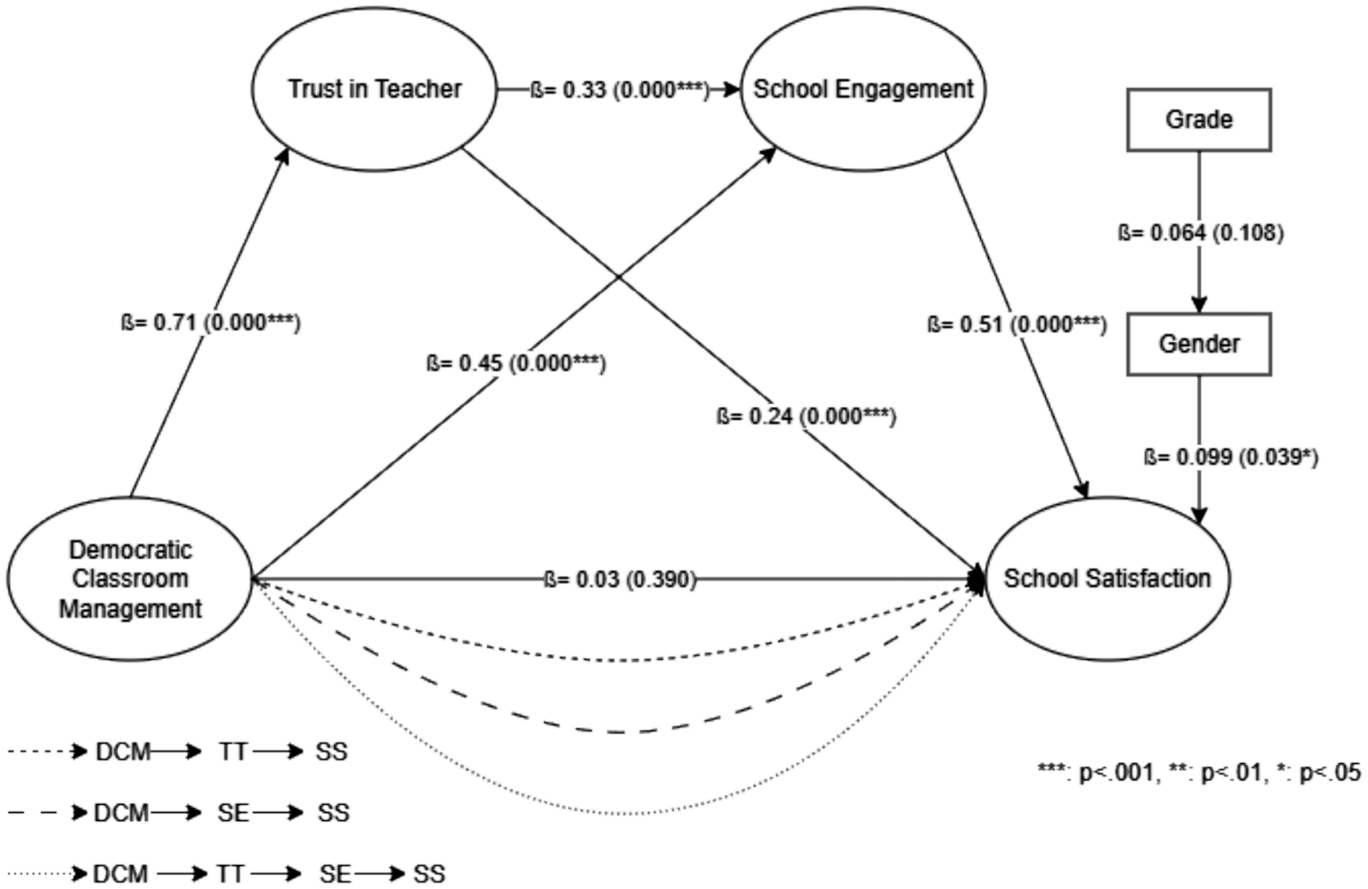
Results of serial multiple mediation SEM analysis of the model.

Following the examination of research models, the Structural Equation Model was conducted using the maximum likelihood estimation to evaluate both direct and indirect mediating relationships between variables (see Figure 2 and Table 6).

**Table 6:** Results of path analysis and regression analysis.

(Dependent variable: TT)	*β*	*p*	Bootstrap 95% CI
Constant	2.17	0.000^***^	2.055, 2.293
DCM	0.71	0.000^***^	0.489, 0.561
*R*^2^ = 0.511. ***p < 0.001, **p < 0.01, *p < 0.05			

(Dependent variable: SE)	*β*	*p*	Bootstrap 95% CI
Constant	1.40	0.000^***^	1.209, 1.588
DCM	0.45	0.000^***^	0.281, 0.381
TT	0.33	0.000^***^	0.258, 0.394
*R*^2^ = 0.523. ***p < 0.001, **p < 0.01, *p < 0.05			

(Dependent variable: SS)	*β*	*p*	Bootstrap 95% CI
Constant	0.32	0.019^*^	0.052, 0.578
DCM	0.03	0.390	−0.038, 0.098
TT	0.24	0.000^***^	0.206, 0.383
SE	0.51	0.000^***^	0.534, 0.705
*R*^2^ = 0.512. ***p < 0.001, **p < 0.01, *p < 0.05			

Direct, indirect and total effects				
Effects	Paths	Effect	*p*	Bootstrap 95% CI
Direct effect	DCM → TT	0.71	0.000^***^	0.489, 0.561
Direct effect	DCM → SE	0.45	0.000^***^	0.281, 0.381
Direct effect	TT → SE	0.33	0.000^***^	0.258, 0.394
Direct effect	TT → SS	0.24	0.000^***^	0.206, 0.383
Direct effect	SE → SS	0.51	0.000^***^	0.534, 0.705
Indirect effect	DCM → TT → SE	0.15	0.000^***^	0.112, 0.225
Indirect effect	DCM → TT → SS	0.17	0.000^***^	0.107, 0.202
Indirect effect	DCM → SE → SS	0.23	0.000^***^	0.163, 0.247
Indirect effect	TT → SE → SS	0.17	0.000^***^	0.151, 0.253
Indirect effect	DCM → TT → SE → SS	0.12	0.000^***^	0.078, 0.134
Direct effect	DCM → SS	0.03	0.390	−0.038, 0.098

DCM, democratic classroom management; TT, trust in teacher; SE, school engagement; SS, school satisfaction. ***p < 0.001, **p < 0.01, *p < 0.05.

Based on Table 6 and Figure 2, the serial multiple mediation model possesses strong explanatory power. Students’ perceptions of DCM account for 51.1% of the Trust in Teacher (TT) variable (*R*^2^ = 0.511); DCM and Trust in Teacher (TT) variables together explain 52.3% of the School Engagement (SE) variable (*R*^2^ = 0.523); and finally, all variables together (DCM, TT, and SE) explain 51.2% of the School Satisfaction (SS) variable (*R*^2^ = 0.512). These results show that the model is theoretically consistent and has strong explanatory power.

#### Direct effects

The findings reveal a strong and positive relationship between DCM and TT (*β* = 0.71, *p* < 0.001); a moderate and positive relationship between DCM and SE (*β* = 0.45, *p* < 0.001); a moderate and positive relationship between TT and SE; a weak and positive relationship between TT and SS; and a moderate and positive relationship between SE and SS. In contrast, no significant relationship was found between DCM and SS. This indicates that the effect of DCM is mediated through indirect effects.

#### Indirect effects

All indirect effects in the model were significant (see Figure 2 and Table 6). Looking at the research results, it can be said that students’ perceptions of DCM first strengthen their trust in the teacher, which in turn strengthens their commitment to the school and consequently increases their satisfaction with the school.

## Discussion

In terms of implications, the current findings can shed some light on the theoretical assumptions of self-determination theory (SDT) and social exchange theory (SET). First of all, regarding the assumption of SDT, the results provide support to the idea that an autonomy-supporting environment satisfies basic needs of students, including the need for autonomy and relatedness, and consequently leads to satisfaction with schools. More precisely, the fact that DCM does not directly predicts school satisfaction but only indirectly affects school satisfaction via the mediators suggests that satisfaction of needs alone is insufficient, and it is rather trust and engagement that make students satisfied with the conditions of studying. The DCM → Trust → Engagement pattern also contributes to the development of SET because, according to this theory, trust-based reciprocal exchanges encourage more social involvement in social institutions. Moreover, none of these variables – socioeconomic status, academic performance, or academic stress – was considered, even though they can serve as potential moderating variables. Previous literature on the subject suggests that children from underprivileged households tend to start out with different levels of trust toward teachers (Fryberg et al., 2013), as well as different levels of engagement with education (Tordön et al., 2021).

It is necessary to take note of the extremely high correlations among the constructs within the model, such as between DCM and trust in teacher (*r* = 0.72) and between school engagement and school satisfaction (*r* = 0.69). The high correlations could imply that the constructs may have some similarities, and this may help to understand why the AVE values were lower than the minimum value of 0.50 for trust in teacher (AVE = 0.467) and school engagement (AVE = 0.411). While the discriminant validity was established due to HTMT ratios being lower than 0.90 (Henseler et al., 2015), there should be caution in interpreting the results due to the overlapping of the constructs.

Within the scope of the first research question of the study, a meaningful, strong, and positive relationship (*β* = 0.71, *p* < 0.001) was found between DCM and trust in teachers, consistent with previous studies conducted in Türkiye (Özer and Karadaş, 2021). Furthermore, the significant association between DCM and trust in teacher (*β* = 0.71) was in line with findings by Peras and Bezjak (2025); however, the current study’s findings indicate a considerably higher effect size compared to that found in previous research done in Europe (e.g., Slovenia). This could be explained by the high importance of teacher power distance in the educational system of countries like Türkiye; that is, because of the great gap between teachers and students, any change in classroom management style would lead to significantly high levels of trust. A moderate positive relationship (*β* = 0.45, *p* < 0.001) was found between DCM and school engagement. In line with this result, Davis et al. (2012) stated in their book that teachers’ positive attitudes toward classroom management enhance students’ school engagement. Furthermore, a study examining research on the websites of organizations focused on developing K-12 schools and education in the United States found that it was concluded that the classroom management approaches most closely related to school engagement are those that support student autonomy and empowerment, reduce social hierarchies and power differences among students, prioritize positive behavior and restorative discipline practices, and emphasize equality and justice (Wilkins et al., 2023). The absence of a significant direct relationship between DCM and school satisfaction (*β* = 0.03, *p* > 0.05) highlights the need to examine this relationship through indirect effects.

In addressing the second research question of the study, Pianta and Stuhlman (2004), in their widely cited study using American elementary school samples, demonstrated that the quality of teacher-child relationships is a significant predictor of children’s engagement and school adjustment in the early school years, highlighting the centrality of relational factors for behavioral and cognitive involvement in school. A more direct parallel is offered by Roorda et al.’s (2011) meta-analysis of 99 studies, predominantly from North American and European contexts. The current finding that trust in teacher mediates the DCM–school engagement relationship (Effect = 0.15, *p* < 0.001) is consistent with these American studies, yet it underscores an important contextual distinction: whereas the aforementioned studies examined the direct relational effects of teacher behavior on engagement, the present study demonstrates that it is specifically the democratic dimension of classroom management that activates trust as the relational bridge to engagement.

Within the context of the third research question, the study demonstrates that trust in teachers and engagement with the school jointly exert a significant effect on school satisfaction, and that perceptions of DCM exert an indirect effect on school satisfaction. This finding demonstrates that the chain of serial mediation is meaningful in the process of students’ perceptions of DCM increasing their school satisfaction (Effect = 0.12, *p* < 0.001). This serial mediation effect shows that students’ perceptions of DCM first increase their trust in the teacher, this trust then strengthens their school engagement, and ultimately increases their school satisfaction. Therefore, this study demonstrates that the concept of DCM plays an indirect but effective role in increasing students’ trust in their teachers, their school engagement, and their school satisfaction. These findings can be interpreted as suggesting that teachers shaping their classroom management skills based on democratic processes will create a learning environment where students feel safe, valued, and involved in the process. Such a classroom climate can increase students’ trust in their teachers while also increasing their sense of belonging to the school and their overall satisfaction with school.

## Conclusion

The study provides empirical support for the indirect effect of DCM on school satisfaction through trust in teachers and school engagement. In line with the postulated tenets of Self-Determination Theory and Social Exchange Theory, it appears that democratic classroom settings are autonomy-supportive and fairness-oriented environments that promote trust and engagement.

The results show that DCM is a strong predictor of trust in teacher and a moderate predictor of school engagement. Nevertheless, its effect on school satisfaction is completely mediated by trust and engagement-based mechanisms. Trust in teacher not only fosters school engagement but also has a direct effect on school satisfaction. School engagement is found to be the strongest predictor of school satisfaction, emphasizing its pivotal role in students’ cognitive and affective appraisals of school experiences.

The results of the serial mediation analyses indicate a developmental sequence: trust is fostered by democratic classroom practices, and this trust enhances engagement, which in turn boosts satisfaction. This sequence illustrates the significance of relational and participatory classroom environments for the promotion of positive school experiences. In competitive educational settings, where students are under pressure to perform and where this performance pressure could jeopardize students’ well-being, DCM could be a protective contextual factor that contributes to the students’ psychosocial development.

The study contributes to the existing body of knowledge by showing that school satisfaction is not only a direct result of classroom practices but also a result of relational and motivational processes. Democratic management practices have a great deal of potential for improving students’ engagement and their overall satisfaction with school experiences.

### Limitations and recommendations for future research

Notwithstanding the contributions, there are a number of limitations in the current study that need to be mentioned. First, the study was conducted using a cross-sectional design, which does not allow for the causal interpretation of the observed relationships. Although the serial mediation model was theoretically informed, longitudinal or experimental research is required to clarify the temporal relationships between DCM, trust, engagement, and satisfaction.

Second, all variables in the current study were assessed using self-report measures, which raises the possibility of shared method variance despite the statistical controls.

Third, while gender and grade level were controlled, other variables that could potentially play a role, such as socioeconomic status, academic achievement, perceptions of academic pressure, or dimensions of classroom climate, were not considered in the model. Future research could consider moderated mediation analyses to determine if the strength of the proposed relationships holds across demographic or contextual variables.

Fourth, the moderately strong correlation among constructs and insufficient AVE values for trust in teachers and school engagement indicate possible redundancy. Although the criteria for composite reliability and HTMT have been met, this issue needs to be kept in mind while evaluating the results of convergent validity.

Future research based on the current findings could investigate reciprocal relationships between engagement and satisfaction, examine the role of teacher autonomy-supportive behavior more specifically, or design intervention studies to improve democratic classroom practices. Such research would help to further elucidate the role of classroom-level processes in shaping students’ academic and psychosocial development.

## Data Availability

The original contributions presented in the study are included in the article/supplementary material, further inquiries can be directed to the corresponding authors.
